# The mediating role of negative symptoms in “secondary factors” determining social functioning in chronic schizophrenia

**DOI:** 10.3389/fpsyt.2023.1196760

**Published:** 2023-08-15

**Authors:** Na Hu, Wei Li, Hu Deng, Jiaqi Song, Hanxue Yang, Jiabao Chai, Wenqian Huang, Hong Wang, Xuanzi Zhou, Pan Zhang, Sushuang He, Yonghua Cui, Tengteng Fan, Ying Li

**Affiliations:** ^1^Beijing Huilongguan Hospital, Peking University Huilongguan Clinical Medical School, Beijing, China; ^2^School of Psychology, Beijing Language and Culture University, Beijing, China; ^3^Fengtai Maternal and Child Health Care Hospital, Beijing, China; ^4^Department of Psychology, Hebei Normal University, Shijiazhuang, China; ^5^Department of Psychiatry, Beijing Children’s Hospital, Capital Medical University, National Center for Children Healthy, Beijing, China; ^6^Peking University Sixth Hospital, Peking University Institute of Mental Health, NHC Key Laboratory of Mental Health Peking University, National Clinical Research Center for Mental Disorders (Peking University Sixth Hospital), Beijing, China

**Keywords:** secondary negative symptoms, chronic schizophrenia, depression, positive symptoms, social functioning

## Abstract

**Background:**

Chronic schizophrenia is significantly influenced by negative symptoms, with several known contributors to secondary negative symptoms. However, the impact of these factors and negative symptoms on social functioning warrants further exploration.

**Methods:**

We assessed the clinical symptoms, antipsychotic adverse reactions, and social functioning of 283 hospitalized patients with chronic schizophrenia using various standardized interviews and scales. We conducted multiple regression and mediation analyses to elucidate the impact of secondary factors on negative symptoms, and the relationship among these “secondary factors,” negative symptoms, and social functioning.

**Results:**

Our findings identified depressive symptoms, extrapyramidal symptoms, and positive symptoms as significant contributors to secondary negative symptoms. We found that negative symptoms play a notable mediating role in the effect of depressive and positive symptoms on social functioning. However, the relationship between positive symptoms, negative symptoms, and social functioning proved to be intricate.

**Conclusion:**

Our findings propose that negative symptoms act as pivotal mediators in the correlation between “secondary factors” (including the depressive symptoms and positive symptoms) and social functioning. The treatment of chronic schizophrenia necessitates focusing on key factors such as depressive and positive symptoms, which might significantly contribute to the development of secondary negative symptoms. Further research is essential to clarify the complex relationship among positive symptoms, negative symptoms, and social functioning in schizophrenia.

## 1. Introduction

Negative symptoms, characterized by deficits in motivation, communication, emotion, and social functioning (1), are considered a core feature of schizophrenia—a chronic and debilitating mental disorder (2). These symptoms significantly deteriorate the functioning and quality of life of patients with schizophrenia (2–5), imposing a substantial burden on patients, their families, and the healthcare system (6). Negative symptoms primarily denote the decrease or absence of typical behaviors (such as indulgence, anhedonia, and social engagement) or expressions (such as emotional dullness, apathy) associated with motivation and interest (7, 8). The current consensus classifies negative symptoms into five domains: Blunted affect, characterized by a reduction in emotional expression, demonstrated by decreased facial and vocal expressions, and physical movements; Alogia, referring to a decrease in the quantity of speech and spontaneous elaboration; Asociality, indicating a diminished drive to engage in interpersonal relationships, leading to reduced social interactions; Anhedonia, describing a decreased capacity to derive pleasure from current activities or anticipate enjoyment from future engagements; Avolition, representing a diminished ability to initiate and maintain goal-oriented activities due to a lack of motivation (9).

Negative symptoms have been differentiated into either primary symptom intrinsic to schizophrenia, or secondary symptom resultant from other underlying factors (6). Primary negative symptoms, considered inherent aspects of schizophrenia’s core pathology, are thus direct manifestations of the disorder (10). Secondary negative symptoms, however, are thought to arise from exogenous factors, they are typically transient and more prevalent than primary negative symptoms (11). Given their clear causative factors, secondary negative symptoms are generally more responsive to treatment compared to primary negative symptoms (11, 12). Secondary negative symptoms in schizophrenia can stem from a variety of sources, including positive symptoms, depressive symptoms, side effects of antipsychotic medication (such as extrapyramidal symptoms), or prolonged social isolation due to extended hospitalization (2, 13–15). According to the European Psychiatric Association (EPA) guidance on assessment of negative symptoms in schizophrenia, there are three key factors that might serve as “secondary factors” which include depressive symptoms, positive symptoms, and extrapyramidal symptoms (7). For instance, extrapyramidal symptoms can lead to a blunted affect and alogia, both of which form part of the negative symptoms (2). However, it remains unclear whether these secondary factors impede social functioning by exacerbating negative symptoms.

Social functioning, defined as the capacity to comprehend and partake in social activities, thus facilitating effective and regular involvement in social life (16), constitutes a crucial facet of the rehabilitation journey for individuals diagnosed with schizophrenia (17). It is important to highlight that impaired social functioning is a typical characteristic of those with schizophrenia, with negative symptoms serving as a major contributor to such deficits (18). Notably, the co-occurrence of depressive symptoms is another influential factor, as it has been found to result in markedly detrimental consequences on social functioning in patients with schizophrenia (19). It is also noteworthy that positive symptoms have been identified as influencing social functioning in patients with this disorder. Furthermore, existing literature has established that patients presenting more severe motor impairments, such as extrapyramidal symptoms, often correspondingly display worsened social functioning (20). Motor abnormalities, such as extrapyramidal symptoms, are believed to result from a combination of brain dysfunction and the side effects of antipsychotic medication (20, 21). Despite these observations, the interrelationships among these secondary factors (which contribute to secondary negative symptoms), negative symptoms themselves, and social functioning call for further exploration.

The main objective of this study is 2-fold. Firstly, we aimed to investigate several potential “secondary factors” that might lead to negative symptoms in chronic schizophrenia. Secondly, by employing mediation analysis, the relationship among (1) negative symptoms, (2) secondary factors (including positive symptoms, depressive symptoms, and extrapyramidal symptoms), and (3) social functioning were explored. For this study, our hypothesis suggests that the “secondary factors” has an impact on social functioning by a mediator of the severity of negative symptoms.

## 2. Materials and methods

### 2.1. Participants

The present study employed a cross-sectional design and conducted a sampling survey at the one of the Mental Health Centers of Beijing from March 1, 2018, to September 30, 2018. A total of 283 hospitalized patients between the ages of 18 and 60, who had been diagnosed with schizophrenia by two psychiatrists according to the fifth edition of the Diagnostic and Statistical Manual of Mental Disorders (DSM-5), were recruited as participants. Eligible participants had a course of schizophrenia lasting more than 5 years and had been hospitalized for at least 5 years.

### 2.2. Inclusion and exclusion criteria

Inclusion criteria of this study were as followed: (1) diagnosed with schizophrenia by the DSM-5 diagnostic criteria; (2) having a disease duration of more than 5 years; (3) hospitalized for longer than 5 years; (4) aged between 18 and 60; (5) had not have any changes in antipsychotic medication for at least 6 weeks; and (6) voluntary participation with informed consent. Exclusion criteria were: (1) having comorbid mental disorders; (2) having brain diseases or serious/unstable physical illnesses; (3) being pregnant or lactating; (4) having drug or alcohol abuse/dependence; (5) having received modified electroconvulsive therapy within the past 3 months; and (6) were unable to complete all surveys. Informed consent obtained from all patients and their guardians. The study protocol was approved by the Ethics Committee of the one Mental Health Center of Beijing (protocol number: 109).

### 2.3. Assessment tools

#### 2.3.1. The positive and negative syndrome scale

The positive and negative syndrome scale (PANSS) is a widely used tool designed to evaluate the severity of schizophrenia symptoms (22). The PANSS has a Chinese version which comprises of 30 items and is divided into three subscales: positive, negative, and general psychopathology. The severity of each item is rated on a Likert scale from 1 (absent) to 7 (extreme), with higher scores indicating more severe symptoms. The Chinese version of the PANSS has been shown to be a reliable and effective assessment tool for evaluating the severity of psychopathology in hospitalized, stable patients with schizophrenia (23).

#### 2.3.2. The scale for assessment of negative symptoms

The scale for assessment of negative symptoms (SANS) is a well-established instrument in evaluating negative symptoms across five domains, including affective flattening, alogia, avolition apathy, anhedonia-asociality, and attention. The scale consists of 25 items, which are rated on a six-point Likert scale ranging from 0 to 5 points, with higher scores indicating greater impairment (24). The Chinese versions of the SANS have good reliability and validity (25).

Factor analysis of the general psychopathological rating scales found that some items in SANS did not belong to the category of negative symptoms, including attentional impairment (SANS global rating of attention), inappropriate affect (SANS item 6), and poverty of content of speech (SANS item 10) (7, 8). Therefore, we have excluded the above three items in the data related to SANS in the article.

#### 2.3.3. The assessment of positive symptoms

The scale for the assessment of positive symptoms (SAPS) scale is comprised of 34 items that evaluate positive symptoms of schizophrenia across four domains: hallucinations, delusions, bizarre behavior, and positive formal thought disorder (24). Each item is scored on a severity scale from 0 (absent) to 5 (extremely severe). Every subscale has its own global score, which ranges from 0 (absent) to 5 (extremely severe), assessing the overall severity of each symptom domain. The SAPS total score is the sum of all items except the global items, while the SAPS summary score is the sum of the four global items. The Chinese version of the SAPS scale has demonstrated good reliability and validity (25).

#### 2.3.4. The Simpson–Angus scale

The Simpson–Angus scale (SAS) is a validated sensitive tool to evaluate extrapyramidal side effects caused by neuroleptic drugs (26), including 10 items that are graded on a five-point Likert scale, with each item scored from 0 to 4. A higher score on the SAS scale indicates the presence of more severe extrapyramidal side effects.

#### 2.3.5. Calgary depression scale for schizophrenia

The Calgary depression scale for schizophrenia (CDSS) was developed to evaluate depressive symptoms in individuals with schizophrenia. It consists of nine items, each rated on a Likert scale of 0–3, with higher scores indicate more severe symptoms (27).

#### 2.3.6. Global assessment of functioning scale

The Global Assessment of Functioning (GAF) is a widely used scale in both research and clinical practice for assessing the overall functioning of patients with schizophrenia, focusing on psychological, social, and occupational well-being (28). The total score ranges from 1 to 100, with higher scores indicating better retained functions and milder symptoms. The GAF is subdivided into 10 10-point intervals. The reliability and validity of the GAF as a valuable objective indicator of overall patient functioning has been strongly suggested (29).

Four professional psychiatrists who had undergone training and consistency evaluation (the Intraclass Correlation Coefficient is 0.90) were recruited to evaluate the schizophrenia scale on site, and the survey results were collected. Each eligible patient was assessed by two psychiatrists on the same day. The first psychiatrist evaluated the patients and collected data on their sociodemographic information, clinical data, and psychiatric history through a questionnaire. The second psychiatrist administered all six scales to the patient.

### 2.4. Data analysis

Data analysis was performed using SPSS for Windows 23.0. Descriptive statistics were used to summarize continuous variables in terms of mean and standard deviation. Firstly, multiple linear regression was used to explore the factors that may significantly impact negative symptoms, with the total SANS score as the dependent variable and the common factors of secondary negative symptoms as independent variables. Secondly, we utilized a linear regression model to separately explore: (1) The prediction of social function as evaluated by Global Assessment of Functioning (GAF), by the extrapyramidal symptoms evaluated by SAS, depressive symptoms evaluated by CDSS, and positive symptoms evaluated by SAPS; (2) The combined prediction of these three factors (SAS, CDSS, and SAPS) for negative symptoms; (3) The prediction of social function (GAF) by negative symptoms, evaluated by Scale for the Assessment of Negative Symptoms (SANS), after excluding items not related to negative symptoms. Furthermore, the impact of each negative symptom factor on social functioning was further studied using multiple linear regression, with the GAF score as the dependent variable and the SANS score on the five subscales as independent variables. Finally, mediation analysis was carried out using packages in R (Mediation) (30) to investigate the relationship between depressive symptoms, extrapyramidal symptoms, positive symptoms, and social functioning, with a focus on the role of negative symptoms. All tests were two-tailed, and the significance level was set at *p* < 0.05.

## 3. Results

### 3.1. Basic information for included patients

A total of 283 long-term hospitalized patients with chronic schizophrenia participated in this study, with an age range of 29–60 and an average age of 43.83 ± 8.19. The mean age of onset was 24.57 ± 8.042, the mean course of disease was 8.92 ± 5.075 years, and the mean length of hospital stay was 6.30 ± 4.535 years. Participants exhibited significant negative symptoms (mean SANS score of 19.548 ± 10.541), and relatively mild positive (mean SAPS score of 3.15 ± 4.466) and depressive symptoms (mean CDSS score of 1.70 ± 2.00). Moreover, their social functioning was impaired, as evidenced by a mean GAF score of 68.16 ± 7.345 (see Table 1).

**Table 1:** Demographic characteristics of the study participants (*N* = 283).

Variables	Mean	SD
Age	43.830	8.190
Education	11.663	2.803
Course of disease	8.920	5.075
Duration of hospitalization	6.300	4.535
Drugs	359.360	171.122
Age of onset	24.570	8.042
PANSS		
PANSS-positive scale	10.120	3.474
PANSS-negative scale	14.890	4.657
PANSS-general psychopathology	24.270	5.045
SAPS	3.150	4.466
SANS	19.548	10.541
SAS	0.820	1.776
CDSS	1.700	2.000
GAF	68.160	7.345

PANSS, Positive and Negative Syndrome Scale; SAPS, Scale for the Assessment of Positive Symptoms; SANS, Scale for the Assessment of Negative Symptoms; SAS, Simpson-Angus Extrapyramidal Symptoms Rating Scale; CDSS, Calgary Depression Scale for Schizophrenia; GAF, Global Assessment Function; SD, Standard Deviation. The drug dose is expressed as the equivalent dose of chlorpromazine.

### 3.2. Multiple linear regression analysis of secondary factors of negative symptoms and social function

The multiple linear regression analysis revealed that negative symptoms, as assessed by the SANS, significantly predict social function as evaluated by the GAF scale (*p* < 0.001). Moreover, the scores from the CDSS (*p* = 0.001), SAS (*p* = 0.007), and the SAPS (*p* = 0.048) significantly predict the SANS score. However, only the CDSS (*p* = 0.019) and SAPS (*p* = 0.045) scores significantly predict the GAF score (see Table 2).

**Table 2:** Multiple linear regression analysis of secondary factors of negative symptoms and social function (*N* = 283).

Variables	Dependent variables	B	S.E.	β	*t*	*p*
CDSS	SANS	1.035	0.934	0.186	3.212	0.001
SAS		0.999	0.365	0.168	2.737	0.007
SAPS		−0.288	0.145	−0.122	−1.982	0.048
CDSS	GAF	−0.538	0.227	−0.139	−2.368	0.019
SAS		−0.214	0.258	−0.052	−0.829	0.408
SAPS		−0.205	0.102	−0.125	−2.001	0.045
SANS	GAF	−0.235	0.039	−0.338	−6.018	0.000

SAPS, Scale for the Assessment of Positive Symptoms; SANS, Scale for the Assessment of Negative Symptoms; SAS, Simpson-Angus Extrapyramidal Symptoms Rating Scale; and CDSS, Calgary Depression Scale for Schizophrenia.

### 3.3. The effect of negative symptoms on social functioning by multiple linear regression analysis

We conducted an extensive investigation into the association between negative symptoms and social functioning. We examined the relationship between the four subscales of SANS and GAF score and found the score in Table 3 (avolition-apathy) predict GAF score (*p* < 0.001; see Table 3).

**Table 3:** The impact of the four subscales of SANS on social functioning by multiple linear regression analysis (*N* = 283).

Variables	B	S.E.	β	*t*	*p*
Age	0.177	0.062	0.198	2.858	0.05
Education	0.234	0.147	0.086	1.595	0.112
Course of disease	0.260	0.110	0.180	2.360	0.019
Age of onset	−0.005	0.069	−0.005	−0.071	0.943
SANSA	−0.261	0.156	−0.155	−1.676	0.095
SANSB	0.292	0.190	0.138	1.538	0.125
SANSC	−1.051	0.185	−0.396	−5.679	0.000
SANSD	−0.024	0.160	−0.011	−0.151	0.880

Dependent variable: GAF score.

SANSA, Subscale of the Negative Symptom Assessment Scale—affective flattening; SANSB, Subscale of the Negative Symptom Assessment Scale—alogia; SANSC, Subscale of the Negative Symptom Assessment Scale—avolition apathy; and SANSD, Subscale of the Negative Symptom Assessment Scale—anhedonia-asociality.

### 3.4. Mediation analysis

**Figure 1:**
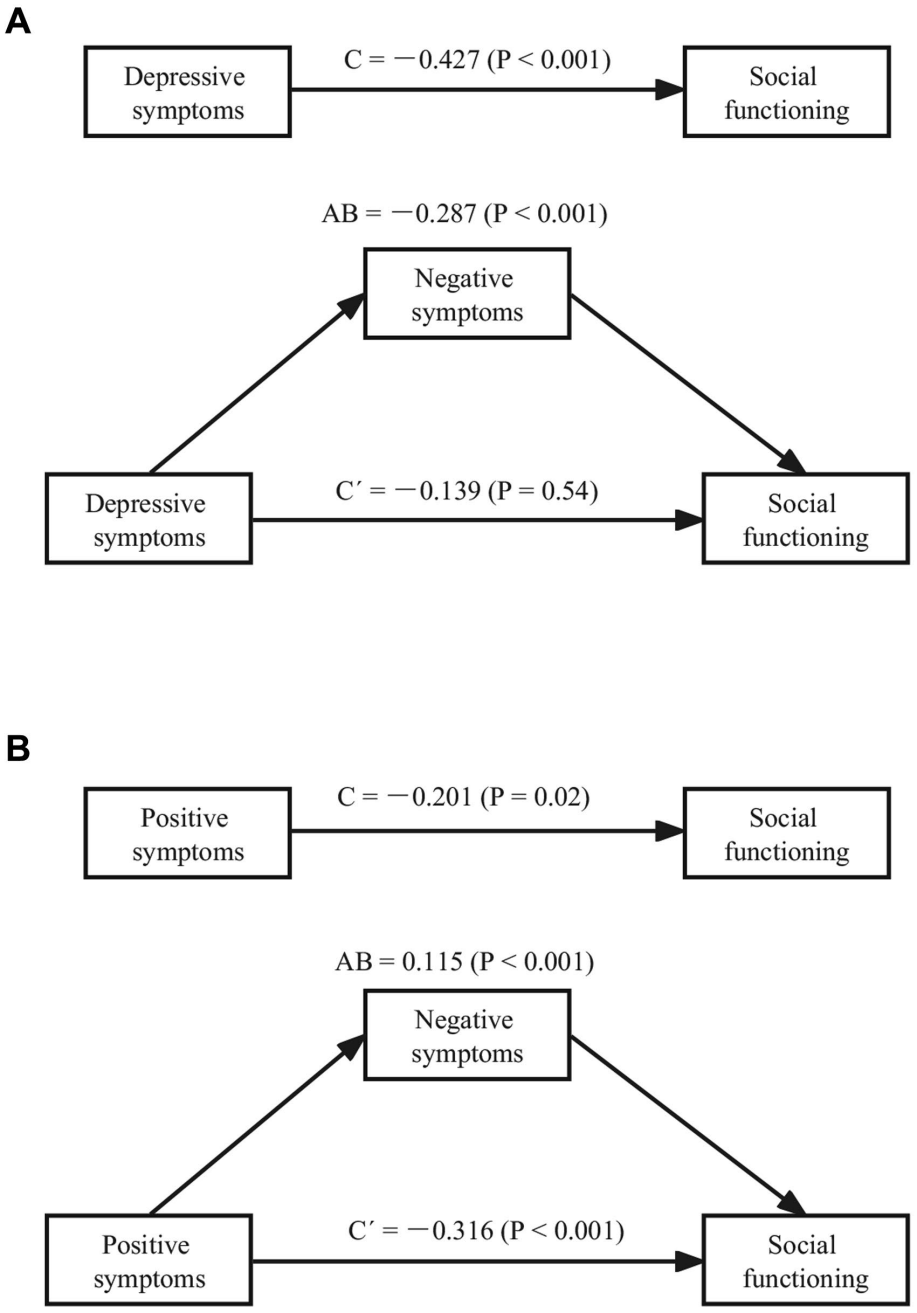
Mediating effect of negative symptoms on depressive symptoms, positive symptoms and social functioning (*N* = 283). **(A)** Mediating effect of negative symptoms on depressive symptoms and social functioning; **(B)** Mediating effect of negative symptoms on positive symptoms and social functioning (C, total effect; AB, indirect effect; C’, direct effect).

In the final stage of data analyses, we investigated the mediating role of negative symptoms in the relationship between various secondary factors and social functioning. Results revealed that negative symptoms fully mediated the relationship between depressive symptoms (β = −0.287, *p* < 0.001) and social functioning, and partially mediated the relationship between positive symptoms (β = 0.115, *p* < 0.001) and social functioning (see Figure 1).

## 4. Discussion

This study aimed to examine the presence of negative symptoms in patients with chronic schizophrenia and to identify factors associated with secondary negative symptoms. Our findings revealed that negative symptoms were significantly associated with positive symptoms and depressive symptoms.

Depressive symptoms are highly prevalent in patients with schizophrenia, occurring in approximately 25% of cases (31), and can manifest at any stage of the disease (11). Moreover, more than 60% of patients with schizophrenia experience depressive symptoms (32), whereas secondary comorbidities of depression are common in chronic schizophrenia (33). Our study confirmed previous findings, which suggest a significant positive correlation between depressive symptoms and negative symptoms in patients with chronic schizophrenia (34). For example, the degree and duration of depressive symptoms in psychiatric patients were correlated with negative symptoms (35).

Furthermore, an overlap between depressive symptoms and negative symptoms in patients with schizophrenia has been noted (11). Anhedonia and avolition are common symptoms of both schizophrenia and depression (36). The key feature of this overlap is anhedonia, which is a core symptom of depression and an important feature of schizophrenia (37, 38). Depressive symptoms are one of the most common factors associated with secondary negative symptoms of schizophrenia (15). Depression can lead to secondary negative symptoms, such as lack of energy, amotivation, and decreased interest (11). Our study also showed that both depressive symptoms and negative symptoms were significantly negatively correlated with GAF scores. A previous study has found that the more prominent the negative and depressive symptoms of schizophrenic patients were, the more severe their impairment of social function (39). In our study, we conducted a mediating analysis and found that negative symptoms completely mediated the relationship between depressive symptoms and social functioning. That is, depressive symptoms had no significant direct effect on social functioning, but mainly influenced it through secondary negative symptoms. Therefore, in the rehabilitation of social functioning in chronic schizophrenia, it is important to pay attention to patients with depressive symptoms, and to intervene possible secondary negative symptoms associated with these depressive symptoms.

Another important factor contributing to negative symptoms being the positive symptoms. Our study found a significant negative correlation between positive and negative symptoms in patients with chronic schizophrenia. However, previous studies have suggested a positive correlation, with improvement of positive symptoms leading to remission of negative symptoms (40). For instance, delusions of victimhood or hallucinations can cause social withdrawal, hindering the patient’s ability to engage in communication or enjoy group activities (34). By treating positive symptoms, such as hallucinations and delusions, negative symptoms secondary to positive symptoms may be alleviated (40). Concerning the discrepancy between our findings and the previous ones, we suspect the characteristics of participants might played a role. All participants in our study were long-term hospitalized patients with chronic schizophrenia, whose negative symptoms were more prominent and positive symptoms relatively mild. When a patient’s condition fluctuates, psychiatrists may increase the dose of antipsychotics, potentially worsening negative symptoms (41). At the same time, decreased positive symptoms may lead to alleviated secondary negative symptoms, which can be relieved by antipsychotics. These results suggest a complex relationship between positive symptoms and secondary negative symptoms that may require further investigation.

Our study results indicate a correlation between extrapyramidal symptoms and negative symptoms. However, these extrapyramidal symptoms do not seem to influence social functioning through negative symptoms. Currently, the relationship between extrapyramidal symptoms and negative symptoms remains unclear. In the two-factor model of negative symptoms, the deficit in the expressive dimension could be affected by extrapyramidal symptoms (2). Therefore, it is plausible that extrapyramidal symptoms could exacerbate the deficit in the expressive dimension of negative symptoms. However, these relationships need further exploration, it is indeed very challenging to differentiate between the Expressive Deficit domain of negative symptoms, which includes blunted affect and alogia (7). Another critical point is the use of antipsychotic medications. The dosage of antipsychotic medications may be a significant factor (7). Future research could focus on exploring the relationship between the dosage of antipsychotic medications, extrapyramidal symptoms, and the deficit in the expressive dimension of negative symptoms.

Additionally, our study found a strong association between apathy, one of the subscales of SANS (24), and social functioning. Apathy is a prevalent negative symptom (42) that significantly affects the social functioning of patients with chronic schizophrenia (43). Our results hinted that reducing apathy should be a priority in the rehabilitation of patients with schizophrenia. Moreover, we proposed that factors leading to secondary negative symptoms might indirectly affect social functioning through negative symptoms. However, our findings indicated that positive symptoms had a direct negative effect on social functioning, consistent with a previous study (44), which reported a significant negative association between positive symptom clusters and social functioning. However, we also found that positive symptoms can positively moderate social functioning by moderating negative symptoms. The relationship between positive symptoms, negative symptoms, and social functioning is complex, and it is affected by drug treatment, side effects, and other factors.

Our study has several clinical implications. First, patients with chronic schizophrenia require special attention and treatment of negative symptoms during social functioning recovery. Among factors contributing to negative symptoms, depressive symptoms and positive symptoms should be the focus of intervention. The intervention of extrapyramidal symptoms should also be considered comprehensively. The treatment process would benefit from controlling depressive symptoms and positive symptoms while minimizing adverse drug reactions, which may aggravate negative symptoms.

Certain limitations in our study merit attention. First, our assessment of negative symptoms solely relied on the SANS and PANSS scales. Future research might benefit from incorporating more recent second-generation negative symptom scales, such as the Brief Negative Symptom Scale (BNSS) and Clinical Assessment Interview for Negative Symptoms (CAINS). These updated scales resonate more with current conceptualizations of negative symptoms and enable a clear distinction between primary and secondary negative symptoms. Secondly, our study lacked data pertaining to the temporal relationship between the onset of negative symptoms and the commencement and augmentation of antipsychotic treatment. As such, we were unable to verify whether all extrapyramidal symptoms we noted were indeed secondary to antipsychotic treatment. Thirdly, considering that not all aspects of function might be impacted by negative symptoms, future research could potentially adopt rating scales that offer a clear separation of functional aspects. This could provide a more nuanced assessment of patients’ social functioning. Fourthly, the relatively small sample size in our study may limit the extrapolation of our findings. To validate our results, larger-scale studies are recommended. Finally, to further unravel the temporal dynamics of negative symptoms and their contributory factors, it would be beneficial for future research to include longitudinal data.

## 5. Conclusion

This study indicates that factors depressive symptoms and positive symptoms may play significant roles in the evolution of secondary negative symptoms in schizophrenia. Our findings propose that negative symptoms act as pivotal mediators in the correlation between “secondary factors” (including the depressive symptoms and positive symptoms) and social functioning. Therefore, in managing chronic schizophrenia, the targeted treatment of depressive symptoms, as well as the positive symptoms could represent a crucial strategy for enhancing social functioning by addressing secondary negative symptoms.

## Data availability statement

The original contributions presented in the study are included in the article/supplementary material, further inquiries can be directed to the corresponding authors.

## Ethics statement

The study protocol was approved by the Ethics Committee of one Mental Health Center in Beijing (protocol number: 109). The studies were conducted in accordance with the local legislation and institutional requirements. The participants provided their written informed consent to participate in this study. Written informed consent was obtained from the individual(s) for the publication of any potentially identifiable images or data included in this article.

## Author contributions

The concept for this article was proposed by YL, and NH, WL, HD, JS, HY, JC, WH, HW, XZ, PZ, SH, YC, and TF collaborated to develop the protocol, establish the search strategy, and define the inclusion and exclusion criteria. Data collection was conducted by NH, WL, HD, JS, HY, JC, WH, HW, PZ, SH, XZ, and YC, while YL performed the statistical analyses, interpreted the data, and created the figures and tables. NH and TF drafted the initial version of the report with critical feedback from YL and YC. NH, WL, HD, JS, HY, JC, WH, HW, XZ, PZ, SH, YC, TF, and YL participated in the interpretation of the findings, and revision of the report, and gave their final approval of the manuscript. All authors contributed to the article and approved the submitted version.

## Funding

This work was supported by the National Natural Science Foundation of China under Grant Nos. 81901345, 82001445, and 82171538, the Natural Science Foundation of Beijing Municipality under Grant Nos. 7212035, and the Beijing Municipal Administration of Hospitals’ Youth Program under Grant No. QML20232006, QML20232003, and QML20211203.

## Conflict of interest

The authors declare that the research was conducted in the absence of any commercial or financial relationships that could be construed as a potential conflict of interest.

## Publisher’s note

All claims expressed in this article are solely those of the authors and do not necessarily represent those of their affiliated organizations, or those of the publisher, the editors and the reviewers. Any product that may be evaluated in this article, or claim that may be made by its manufacturer, is not guaranteed or endorsed by the publisher.
